# Formation of Neutrophil Extracellular Traps by Reduction of Cellular Cholesterol Is Independent of Oxygen and HIF-1α

**DOI:** 10.3390/ijms23063195

**Published:** 2022-03-16

**Authors:** Timo Henneck, AhmedElmontaser Mergani, Sabrina Clever, Anna E. Seidler, Graham Brogden, Sandra Runft, Wolfgang Baumgärtner, Katja Branitzki-Heinemann, Maren von Köckritz-Blickwede

**Affiliations:** 1 Department of Biochemistry, University of Veterinary Medicine Hannover, 30559 Hannover, Germany; timo.henneck@tiho-hannover.de (T.H.); ahmed.mohamed@tiho-hannover.de (A.M.); sabrina.clever@tiho-hannover.de (S.C.); seidler.anni@gmx.de (A.E.S.); graham.brogden@tiho-hannover.de (G.B.); katja.branitzki-heinemann@tiho-hannover.de (K.B.-H.); 2 Research Center for Emerging Infections and Zoonoses (RIZ), University of Veterinary Medicine Hannover, 30559 Hannover, Germany; 3 Department of Pathology, University of Veterinary Medicine Hannover, 30559 Hannover, Germany; sandra.runft@tiho-hannover.de (S.R.); wolfgang.baumgaertner@tiho-hannover.de (W.B.)

**Keywords:** neutrophil extracellular traps, hypoxia, statin, HIF-knock-out mice

## Abstract

Formation of neutrophil extracellular traps (NETs) is a two-faced innate host defense mechanism, which, on the one hand, can counteract microbial infections, but on the other hand, can contribute to massive detrimental effects on the host. Cholesterol depletion from the cellular membrane by Methyl-β-cyclodextrin (MβCD) is known as one of the processes initiating NET formation. Since neutrophils mainly act in an inflammatory environment with decreased, so-called hypoxic, oxygen conditions, we aimed to study the effect of oxygen and the oxygen stress regulator hypoxia-inducible factor (HIF)-1α on cholesterol-dependent NET formation. Thus, murine bone marrow-derived neutrophils from wild-type and HIF-knockout mice or human neutrophils were stimulated with MβCD under normoxic (21% O_2_) compared to hypoxic (1% O_2_) conditions, and the formation of NETs were studied by immunofluorescence microscopy. We found significantly induced NET formation after treatment with MβCD in murine neutrophils derived from wild-type as well as HIF-1α KO mice at both hypoxic (1% O_2_) as well as normoxic (21% O_2_) conditions. Similar observations were made in freshly isolated human neutrophils after stimulation with MβCD or statins, which block the HMG-CoA reductase as the key enzyme in the cholesterol metabolism. HPLC was used to confirm the reduction of cholesterol in treated neutrophils. In summary, we were able to show that NET formation via MβCD or statin-treatment is oxygen and HIF-1α independent.

## 1. Introduction

Neutrophils are the well-known first-line defenders of the innate immune system, which migrate to the site of infection and use a set of different antimicrobial actions against invading pathogens. The formation of neutrophil extracellular traps (NETs) has drawn a lot of attention since their discovery [1]. Meanwhile, it is well described that NETs are formed in response to bacterial [2], fungal [3], parasitical [4], and also viral infections [5,6]. Despite their host defending characteristics, over the years, more and more evidence has accumulated, showing detrimental effects of NET formation in diseases like thrombosis [7], autoimmune diseases, such as lupus [8,9], or cystic fibrosis [10,11]. It is apparent that overshooting NET formation and insufficient clearance of NETs play a crucial role during acute respiratory distress syndrome (ARDS), acquired for example, by severe influenza [6,12] or SARS-CoV-2 infections [13,14]. Without a doubt, neutrophils and their extracellular traps are involved in a variety of actions in diseases of different origins. However, despite their substantial role in these processes, the mechanism of NET formation is yet to be fully understood in all its facets. The literature describes NET formation to be distinct from necrosis and apoptosis [15], requiring specific cellular processes such as reactive oxygen species (ROS) dependent disruption of the nuclear membrane, and mixing of nuclear components and cytoplasmic granules, a process called NETosis. Nonetheless, NETosis can be initiated by different enzymes like myeloperoxidase (MPO), neutrophil elastase, and peptidyl arginases (PAD), which lead to histone degradation and decondensation of the nucleus [15,16,17]. In addition, cholesterol, as an important molecule for cellular membrane composition and signaling, was shown to be involved in the process of NET formation, since its depletion from the cellular membrane by MβCD led to strong NET formation associated with the death of the cell, a phenomenon called NETosis [18,19]. Nevertheless, the role of oxygen in this process remains to be elucidated. The widely used stimulation of NET formation in vitro via PMA (Phorbol-12-myristat-13-acetat) was shown to be not only ROS dependent, but also to require the presence of dissolved oxygen in the surrounding medium [20], which highlights the necessity to involve oxygen as a factor of interest during investigation of cellular processes. Most in vitro experiments are carried out under atmospheric oxygen levels of 21% (normoxia), despite the fact that already in the healthy host, these oxygen levels are not reached in vivo in the different tissues [21,22,23]. Moreover, infection sites may show strongly reduced oxygen levels (hypoxia) due to high oxygen demand of active immune cells. Therefore, we performed NET formation assays in this study under normoxic (18–21% O_2_) and hypoxic (1% O_2_) conditions in parallel, to also assess the influence of oxygen on cholesterol-dependent NET formation. Additionally, we were interested in the role of HIF-1α as oxygen-dependent cellular regulator, which we addressed with a mouse breed that showed reduced HIF-1α function in the myeloid cell linage due to cutting off the *hif-1α* exon 2 by a heterozygously expressed Cre-recombinase [24,25]. HIF-1α is a cytosolic protein, which is continuously degraded under the presence of oxygen due to post-translational modification via hydroxylation and ubiquitination. However, without oxygen, HIF-1α is stabilized, transmigrates to the nucleus where it dimerizes with the HIF-1β subunit and functions as a transcription factor [26,27]. Interestingly, a link between NET formation and the activity of HIF-1α is found in the literature [28,29]. Including these aspects, we wanted to investigate the role of oxygen and HIF-1α on the cholesterol depletion derived NET formation as described by Neumann et al., 2014a. Thus, for this study, we investigated freshly isolated neutrophils of murine origin from wild-type compared to HIF-1α-deficient mice for their NET formation behavior in response to MβCD under both normoxia and hypoxia. Moreover, we investigated NET formation and cellular cholesterol levels in human neutrophils after treatment with MβCD or statins under altering oxygen conditions.

## 2. Results

### 2.1. Establishment of Hypoxia Conditions and HIF-1α-Deficient Neutrophils to Study NET Formation

**Figure 1:**
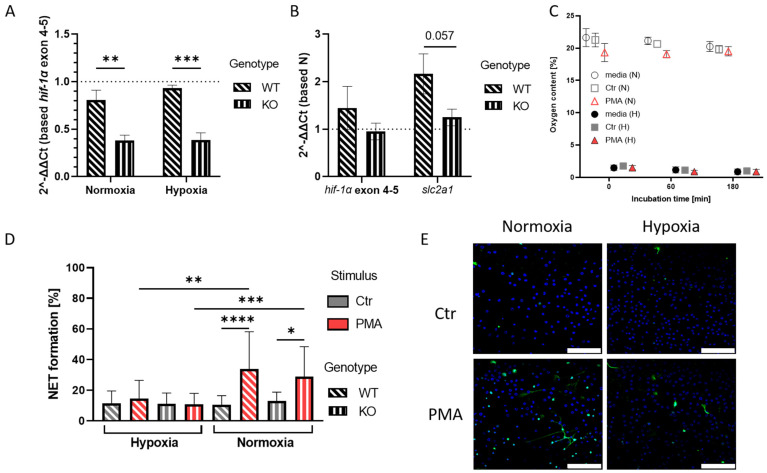
(**A**): The graph shows fold change of *hif-1α* exon 2 expression normalized to *hif-1α* exon 4-5 expression in KO mice (slanted dashed pattern). The KO shows expression reduced by 42% under normoxia and 55% under hypoxia, compared to the WT samples (straight dashed pattern). Significance was analyzed via 2-way-ANOVA with multiple comparisons. (**B**): Here, the fold change of *hif-1α* exon 4-5 expression and HIF-1α target gene *slc2a1* is shown in untreated hypoxia samples, which were normalized to expression levels under normoxia. No upregulation of HIF-1α target gene in KO samples can be observed. Significance was analyzed via the Man–Whitney test. (**C**): Oxygen levels in hypoxia and normoxia samples during 3 h incubation revealed that PMA stimulation under normoxia led to slightly reduced oxygen levels. Overall, sample oxygen levels remained stable during the incubation time. (**D**): NET formation analysis after 3 h incubation of neutrophils under hypoxia or normoxia. Under normoxia, PMA induced NET formation in both WT and KO cells, while no NET formation was observed under hypoxia. Significance was analyzed via 1-way-ANOVA with multiple comparisons. (**E**): Representative immune fluorescence images of NET formation under different oxygen conditions in the WT mice. Left images show NET formation under normoxia, right images show NET formation under hypoxia, with inhibited NET formation after PMA treatment under hypoxia. Blue: DAPI, green: DNA/Histone1 complexes. Scale bar: 100 µm. N = 3/4 (WT/KO) for all shown data sets. (* *p* < 0.05, ** *p* < 0.01, *** *p* < 0.001, **** *p* < 0.0001).

To study the role of HIF-1α in the MβCD-induced NET formation, we isolated bone marrow-derived neutrophils via antibody-dependent negative selection from wild-type and HIF-1α-deficient mice [24,25]. The purity of isolated cells was confirmed by flow cytometry (Supplementary Material, Supplementary Figure S1). As a control experiment to confirm HIF-1α-deficiency, we investigated the expression of *hif-1α* exon 2 in comparison to exon 4–5 expression. We confirmed a significantly reduced transcript expression by around 50% of the targeted exon 2 in the knockout mice (KO) under both normoxic (N) and hypoxic (H) conditions (Figure 1A). Additionally, the transcript expression of the HIF-1α target gene *slc2a1*, a glucose transporter, was examined via RT-qPCR. We could show that the expression of *slc2a1* was distinctly (*p* = 0.057) increased in the wild-type (WT) mice under hypoxia relative to normoxic expression levels, whereas the expression in the KO mice remained unchanged compared to normoxia (Figure 1B). These data confirm that this mouse model is appropriate to study the effect of HIF-1α in neutrophils.

To study NET formation under altering oxygen levels, a continuous application of hypoxic conditions was necessary. Via non-invasive measurement of oxygen levels in the medium, we were able to confirm that the hypoxic conditions were applied over the course of the experiment. The oxygen levels measured under normoxic conditions remained unchanged at 19–21% O_2_, while the samples incubated under hypoxia showed low levels of oxygen of 1–1.5% O_2_ (Figure 1C). PMA treatment of cells was used as a control since PMA is known to efficiently induce NET formation in an oxygen-dependent manner [20]. Using immunofluorescence microscopy, we quantified NET-formation in control versus PMA-stimulated cells and confirmed that PMA triggered NET formation in the bone marrow-derived mouse neutrophils after 3 h stimulation. Additionally, we could show that PMA-induced NET formation in this murine model was significantly inhibited under hypoxic conditions in both WT and KO, which goes in line with the findings described for human neutrophils in a previous study [20]. We did not observe a significant difference in NET formation upon PMA treatment under normoxia or hypoxia between wild-type and HIF-1α-deficient mice, indicating that HIF-1α does not influence this NET formation pathway initiated by PMA (Figure 1D,E).

In summary, the initial control experiments show that the model is functionally valid to study the effect of oxygen and the oxygen stress regulator HIF-1α on cholesterol-dependent NET formation.

### 2.2. NET Formation via Cholesterol Depletion Is Independent of HIF-1α Expression in Murine Neutrophils and Independent of Oxygen

**Figure 2:**
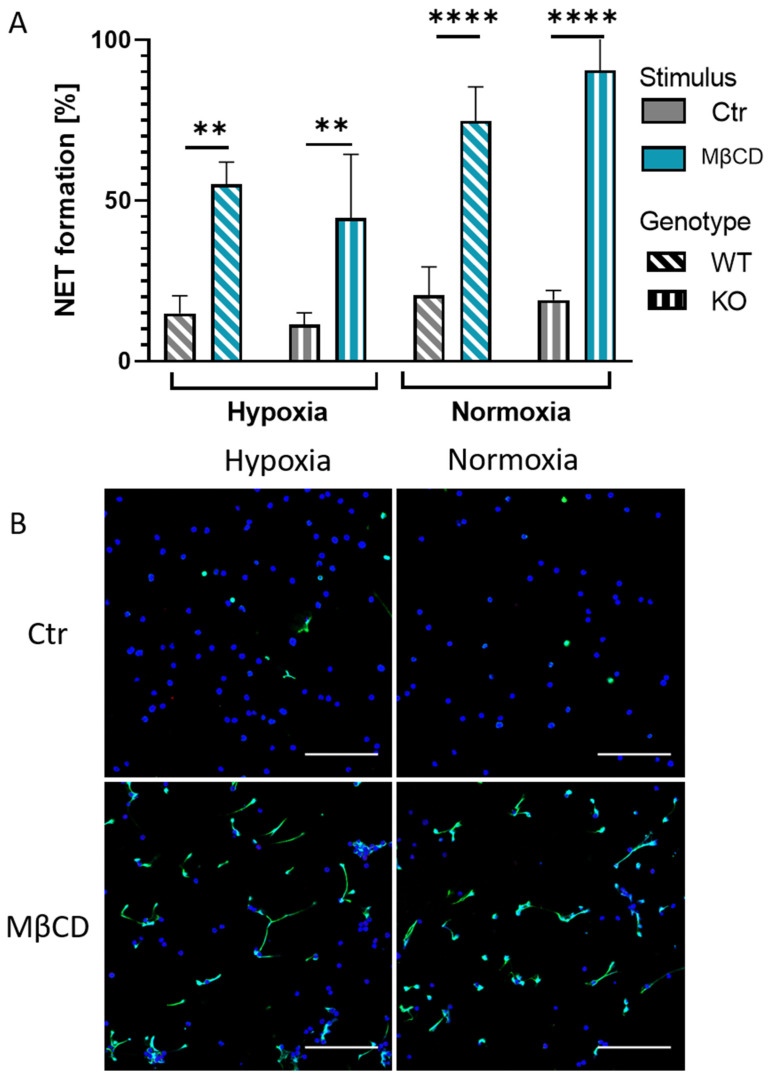
(**A**): Murine neutrophils of WT and KO mice were stimulated with cholesterol-depleting agent MβCD for 3 h under normoxia and hypoxia. Neither low oxygen conditions nor the genotype of the mice had a significant influence on the rate of NET formation. Significance was analyzed via the Kruskal–Wallis test with multiple comparisons. (**B**): Representative immune fluorescence images of KO mice. Left side, hypoxia incubated neutrophils after control or MβCD stimulation. Right side, normoxia incubated neutrophils after control or MβCD stimulation. Blue: DAPI, green: DNA/Histone1 complexes. Scale bars: 100 µm. N = 3. (** *p* < 0.01, **** *p* < 0.0001).

The formation of NETs by depletion of cholesterol via MβCD is well described [18]. Thus, we wanted to know if this mechanism is altered under hypoxia or influenced by the function of HIF-1α. Therefore, we isolated neutrophils from the murine bone marrow of WT and KO mice, incubated them with MβCD for 3 h, and subsequently evaluated the rate of NET formation via immune fluorescence microscopy. We could show that MβCD induced NET formation to a high extent in murine neutrophils (Figure 2) under normoxic as well as hypoxic conditions in both genotypes. Altering oxygen conditions did not lead to significant differences in NET formation values (Figure 2A) within the genotypes (MβCD hypoxia vs. MβCD normoxia, *p* ≥ 0.99 for WT, *p* = 0.07 for KO). Moreover, also between WT and KO, no significant differences were observed when the cells were stimulated (hypoxia: WT vs. KO *p* ≥ 0.99, normoxia: WT vs. KO *p* ≥ 0.99). Figure 2B shows representative images of NET formation induced by MβCD in the KO mice under both oxygen conditions, where it is observable that NET formation efficiently occurs under hypoxia as well as normoxia. These findings led to the hypothesis that NET formation via cholesterol depletion happens in a HIF-1α independent manner. To further investigate the influence of oxygen and to confirm the findings from the murine model, we repeated the experiments in the human model with peripheral blood-derived neutrophils.

### 2.3. NET Formation via Cholesterol Depletion Is Oxygen Independent in Human Neutrophils

**Figure 3:**
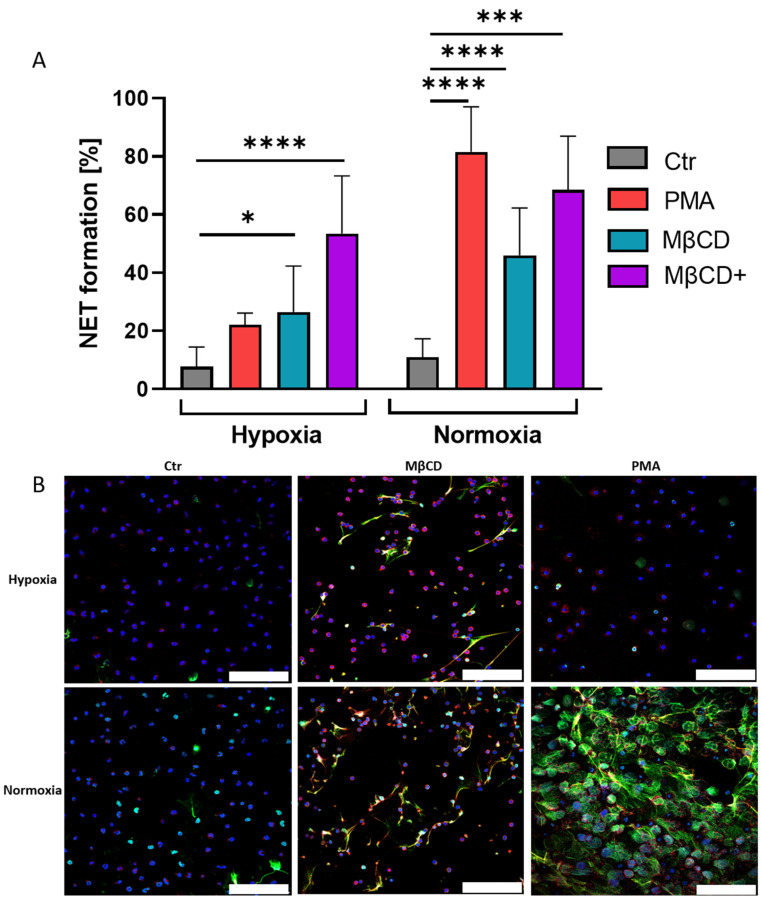
(**A**): Human neutrophils incubated under hypoxia and normoxia after stimulation with either PMA, MβCD, or MβCD+. NET formation by PMA was inhibited under hypoxia, MβCD+ stimulation resulted in NET formation under hypoxia and normoxia. Significance was analyzed via 1-way ANOVA with multiple comparisons. (**B**): Representative immune fluorescence images. The upper row shows NET formation with respective stimulus under hypoxia, bottom row for normoxia. Blue: DAPI, green: DNA/Histone1 complexes, red: myeloperoxidase (MPO). Scale bars: 100 µm. N = 4, (=3 for MβCD+). (* *p* < 0.05, *** *p* < 0.001, **** *p* < 0.0001).

As the following step, we performed NET assays with neutrophils freshly isolated from peripheral human blood to see if MβCD induced NET formation under hypoxia and normoxia in a similar manner in human cells. We triggered NET formation in human neutrophils by both stimuli, PMA as control substance, and MβCD (Figure 3). As expected, we observed the inhibition of PMA induced NET formation under hypoxia (Figure 3A), whereas PMA efficiently induced NETs under normoxia. The stimulation by MβCD with 10 mM resulted in a significant NET-induction under normoxia as well as hypoxia. When comparing hypoxia with normoxia, a tendency for a reduced rate of NET formation under hypoxia is seen, but not statistically significant (*p* > 0.73). Since MβCD-dependent increase of NET formation is concentration-dependent [18], we chose to include a two-fold increased concentration of MβCD in the human NET formation assays of 20 mM (MβCD+) to allow more efficient or faster cholesterol depletion. With higher concentrations of MβCD, NET formation increased even higher, reaching similar levels in hypoxia and normoxia. In summary, we could observe that hypoxic and normoxic neutrophils, stimulated with MβCD+, showed strong significance in NET formation under both hypoxia and normoxia (Figure 3A), supporting the findings of the murine model, suggesting an oxygen-independent NET formation pathway by cholesterol depletion.

### 2.4. Statins Induce NET Formation in Human Neutrophils in an Oxygen Independent Manner

Finally, we used statins as they are a physiologically relevant NET inducer via the manipulation of cellular cholesterol. Statins are a set of drugs widely used especially in the elderly population, as a treatment to reduce cholesterol levels and were already shown to induce NET formation [19]. In our experiments, we used the statins Simva- and Mevastatin as solitary stimuli.

**Figure 4:**
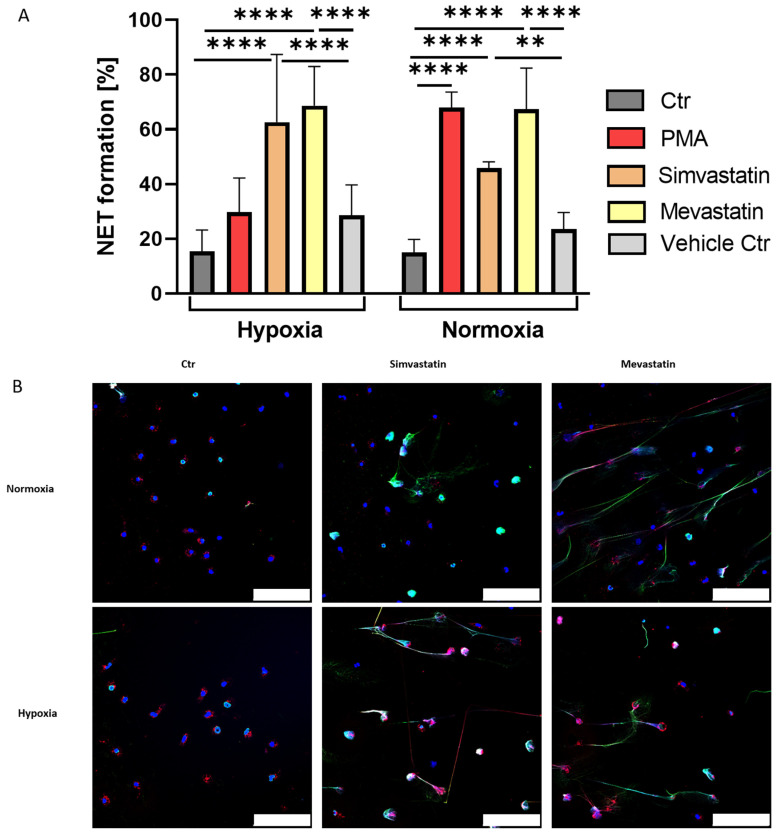
(**A**): Simvastatin and Mevastatin strongly induce NET formation in human neutrophils under hypoxia and normoxia. PMA showed the already described oxygen dependency, while both statins did not differ significantly between the altering oxygen conditions. Significance was analyzed via 1-way ANOVA with multiple comparisons. (**B**): Representative immune fluorescence images of statin-induced NET formation under hypoxia and normoxia, as well as respective negative controls. Both statins showed a similar phenotype of long distinct NET fibers under both oxygen conditions. In addition, both stimuli showed strong positive staining for myeloperoxidase (MPO). Blue: DAPI, green: DNA/Histone1 complex, red: MPO. Scale bars: 100 µm. N = 3. (** *p* < 0.01, **** *p* < 0.0001).

**Figure 5:**
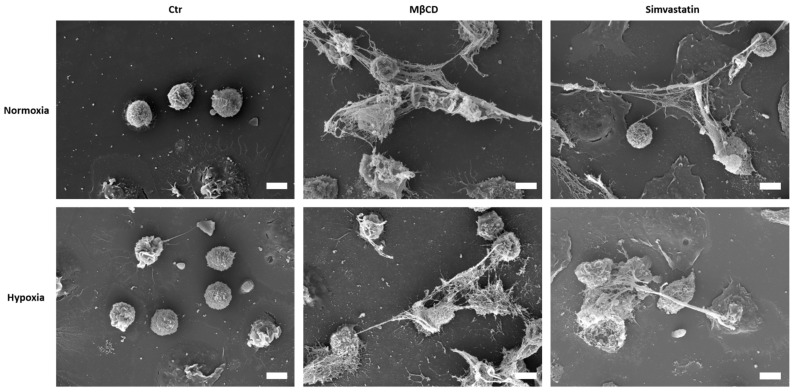
Scanning electron micrographs of human neutrophils, which were incubated under hypoxic or normoxic conditions for 3 h with respective stimuli. Under both oxygen conditions, NET formation can be observed for MβCD and Simvastatin stimulated cells, with typical offshoots. Scale bar: 10 µm.

Freshly isolated neutrophils were incubated for 3 h with Simva- and Mevastatin to investigate NET formation and the influence of oxygen. Again, PMA served as positive and oxygen-dependent control. We found that both statins alone were able to trigger NET formation (Figure 4A). As expected, PMA induced NET formation in an oxygen-dependent manner, with high levels of NETs under normoxia and strongly reduced levels under hypoxia. Both statins significantly induced higher NET formation compared to negative control and vehicle control samples, showing their respective capability to induce NET formation alone and to a similar extent as PMA or MβCD (Figure 3A and Figure 4A). Thereby, the phenotype of NETs induced by statins was observed to be similar to what was seen for MβCD induced NET formation with long, fine NET fibers, observable by immune fluorescence microscopy as well as on single-cell level via scanning electron microscopy (Figure 3B, Figure 4B and Figure 5). Importantly, we could show that NET formation rates between neutrophils stimulated with Simvastatin (*p* = 0.11) or Mevastatin (*p* > 0.99) did not differ significantly between hypoxia and normoxia. Thus, we conclude that both effects of Simva- and Mevastatin are oxygen-independent, which goes in line with similar data shown for MβCD(+).

### 2.5. MβCD Leads to Strongly Reduced Cellular Cholesterol Levels in Murine Bone Marrow-Derived as Well as Human Blood-Derived Neutrophils after 3 h, Independent of Oxygen Level

**Figure 6:**
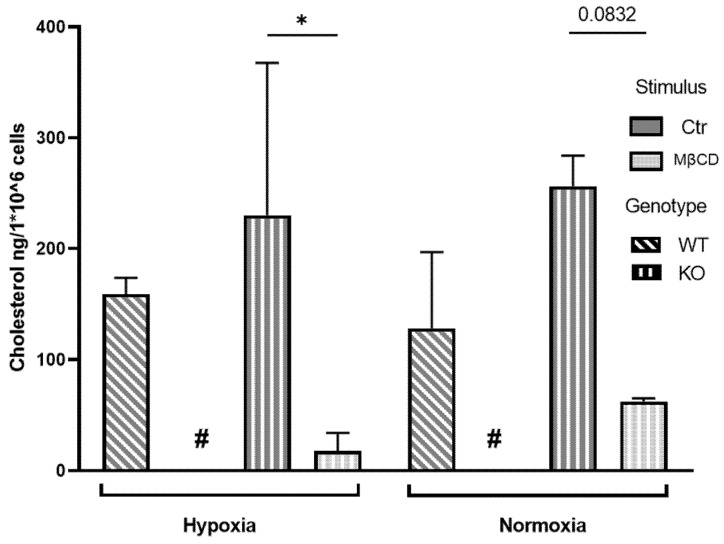
Cellular cholesterol levels of bone marrow-derived murine neutrophils after incubation under hypoxia. In the KO samples, MβCD stimulation resulted in a significant reduction of the cellular cholesterol level. Cellular cholesterol levels after incubation under normoxia revealed a tendency to reduce cholesterol levels in the KO after MβCD treatment, “#” indicates values were below the detection limit of 10 ng thus statistical analysis was not possible for these samples. However, a strong reduction of cellular cholesterol levels seems apparent. Statistical analysis was performed via mixed-effect-analysis with subsequent multiple comparisons. N = 3. (* *p* < 0.05).

**Figure 7:**
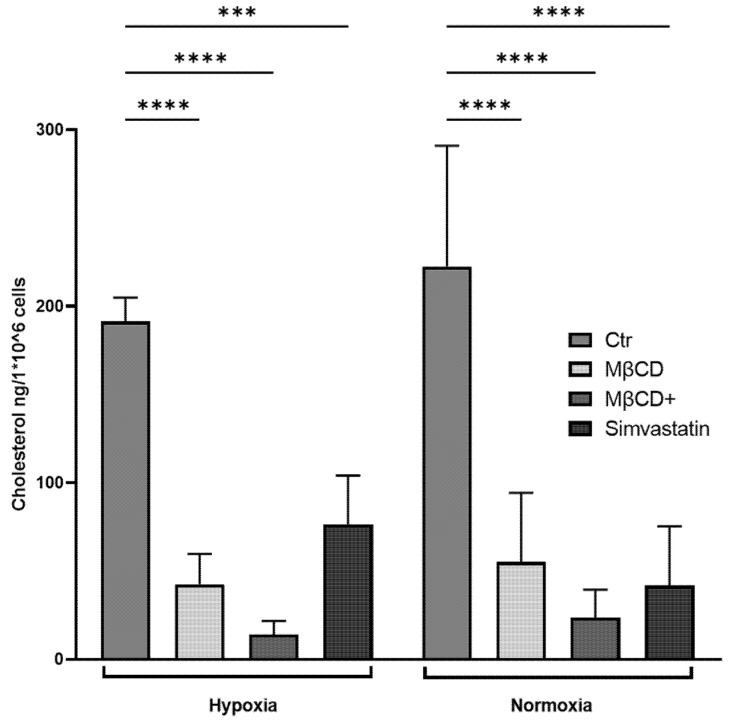
Cholesterol levels of human neutrophils after stimulation with indicated reagents and incubation under hypoxia for 3 h. All stimuli significantly reduced cellular total cholesterol levels within the incubation time. Cellular cholesterol levels after incubation under normoxia were significantly reduced by all stimuli. Statistical analysis was performed via mixed-effect-analysis with subsequent multiple comparison. N = 4. (*** *p* < 0.001, **** *p* < 0.0001).

As a final step, we measured cellular cholesterol levels in murine and human neutrophils after respective stimulation with either MβCD or statins and incubation under normoxia or hypoxia to confirm the actual reduction of cholesterol levels. We incubated neutrophils isolated from the bone marrow of WT and KO mice for 3 h with either medium or MβCD under normoxic or hypoxic conditions. Afterward, lipids were extracted and quantified via High Performance Liquid Chromatographie (HPLC) for changes in total cellular cholesterol (Figure 6 and Figure 7). We could show in the KO mice that MβCD significantly reduced cellular cholesterol levels under hypoxia while normoxia samples revealed a tendency to reduce cholesterol levels after treatment (Figure 6). In the WT samples, a statistical determination was not feasible since cholesterol levels were below the limit of detection of 10 ng (indicated by # in Figure 6) in all 3 samples of WT cells, stimulated with MβCD under hypoxia and 2/3 of the samples under normoxia. However, a reduction of cholesterol levels below the limit of detection is a clear sign of the strong and oxygen independent activity of MβCD in these samples. Moreover, murine neutrophils did not show significant differences between hypoxic or normoxic conditions (MβCD hypoxia vs. MβCD normoxia, *p* = 0.97 for KO) and genotypes (Ctr hypoxia: WT vs. KO *p* = 0.78, Ctr normoxia: WT vs. KO *p* = 0.26). These data suggest that cholesterol depletion via MβCD works independently of oxygen and HIF-1α in murine neutrophils.

Similarly, in the human model, we could additionally confirm that MβCD, MβCD+, and Simvastatin were able to significantly reduce the cellular cholesterol content under both oxygen conditions (Figure 7).

## 3. Discussion

The formation of NETs is mainly initiated as a reaction of the innate immune system to an ongoing infection and mediating entrapment of pathogens. New evidence shows that NETs efficiently boost the phagocytosis of bacterial pathogens by macrophages [30]. However, as described in the introduction, NETs can cause severe damage to host tissue and worsen the outcome of different diseases. Cholesterol is an important membrane component and involved in numerous cell signaling pathways as well as alterations of cellular functions, such as NET formation [18]. At the site of infections, neutrophils act under reduced oxygen conditions in the inflammatory environment due to increased oxygen consumption by the active cells and insufficient oxygen supply [31,32]. Thus, it is important to investigate neutrophil behavior under respective oxygen conditions. To conduct this, we chose a set of approaches to investigate whether hypoxia itself and HIF-1α have an influence on cholesterol-dependent NET formation. Therefore, in this study, we aimed to clarify the following questions: (I) is HIF-1α involved in NET formation triggered via cholesterol depletion? (II) Is cholesterol depletion triggered NET formation independent of oxygen?

To answer these questions, as a first step, we showed that we had established a setup for hypoxic in vitro experiments with murine and human neutrophils. We were able to monitor the oxygen values over the course of the experiment to ensure the application of hypoxic conditions over all time points of the experiment (Figure 1C). This establishment was necessary for bringing the in vitro settings one step closer to the actual in vivo situation and thereby enabling us to set our data in closer contact with the processes in the living host.

At a first instance, we were able to show for the first time that neutrophils isolated from murine bone marrow showed the same phenotype of oxygen dependence when stimulated with PMA to release NETs, as it was described for human neutrophils [20]. We observed that murine neutrophils stimulated with PMA released NETs under normoxia but not under hypoxia and independent of HIF-1α (Figure 1D).

Next, we were interested in the role of cholesterol on NET formation under hypoxia. Thus, we performed NET assays with cholesterol-depleting agent MβCD under hypoxia and normoxia with neutrophils of WT and KO mice, which showed independence of HIF-1α expression and oxygen content (Figure 2A). For further confirmation, we went over to the human model, where we, on the one hand, reproduced and confirmed findings on the oxygen dependency of PMA stimulated NET formation in human neutrophils (Figure 3A) [20] and, on the other hand, showed that cholesterol depletion triggered NET formation in an oxygen-independent manner (Figure 3A). Statins, a group of drugs widely used in human medicine for the treatment of hypercholesterolemia, were used as the physiological stimulus of high relevance to confirm the findings of the previous experiments since statins inhibit the activity of HMG-CoA-reductase, a rate-limiting enzyme in cholesterol biosynthesis. It was shown in previous studies that these drugs are able to efficiently induce NET formation when they were paired with PMA. At the same time, statin treatment reduced the overall production of reactive oxygen species (ROS) in neutrophils, suggesting that statins may predispose cells to enter the NET cell death pathway in response to a lower threshold level of ROS signal [19]. Here, in this study, we investigated whether statins alone were able to induce relevant NET formation independent of available oxygen. Our data showed that human neutrophils at 1% oxygen still exhibit efficient NET formation as a response to statin treatment (Figure 4A). Thus, we conclude that NET formation via statins and MβCD(+) works oxygen-independent in human neutrophils. However, it remains to be elucidated how exactly statins and MβCD can induce NET formation. MβCD rapidly removes large amounts of cellular cholesterol mainly from the cell surface, and at longer time points (>120 min) also from intracellular compartments, such as recycling endosomes and/or late endosomes/lysosomes [33,34]. Reduced cell viability due to strong cholesterol depletion should be considered, but it seemed not problematic in this study, as there were still intact nuclei with typical respective staining for MPO in intact granules, visible during immune fluorescence microscopy (Figure 3B). Moreover, it was described that MβCD treatment of HL-60 cells did not reduce cell viability when treated with 20 mM MβCD for 45 min [33].

We could show in the murine and human model (Figure 6 and Figure 7) that MβCD treatment results in a strong reduction of cholesterol after 3 h and that this effect appears in both hypoxic and normoxic conditions. This leads to the conclusion that MβCD depletes cholesterol from cellular membrane independent of the oxygen level and is more efficient at higher concentrations (MβCD+, Figure 7). In contrast, it is well known that statin-mediated inhibition of cholesterol synthesis will require several days to manifest in significantly lower cellular cholesterol levels. We expected that a 3 h statin incubation would most likely just block the flux of newly synthesized cholesterol into the ER-pool of cholesterol, which makes only 1–2% of total cellular cholesterol. Surprisingly, Simvastatin treatment resulted in a significant reduction of cellular cholesterol levels in the human neutrophils under both hypoxia and normoxia. To our knowledge, this phenotype was not yet described in neutrophils. However, possible additional off-target effects independent of cholesterol alterations cannot be excluded for the statin-treated group and need to be additionally evaluated in future work.

Furthermore, it remains to be determined if the statin or MβCD-mediated NET-formation is associated with NETosis or if a vital pathway of NET formation is involved. During vital NET formation, in contrast to the suicidal NET formation, the cell releases NETs quickly and remains mostly intact by extruding NETs into the extracellular space via small vesicles, while the cell itself remains able to perform other functions, such as phagocytosis [35,36]. Interestingly, based on a recent proteomic analysis, it was described that NETs released in response to different stimuli are heterogeneous in its appearance [37]. Thus, future work should aim to characterize the detailed mechanisms and subsequent composition of NETs under hypoxia versus normoxia.

Independent of the mode of action or efficiency of the cholesterol alteration in the cell, this study shows that NET formation can occur via oxygen independent pathways (Figure 2A, Figure 3A and Figure 4A). Thus, NET formation can contribute to the severity of the disease associated with hypoxia, e.g., infections and inflammation, even though other studies highlight the need for ROS and thus the presence of oxygen for NET formation [15,16,38]. In the context of the recent pandemic situation due to SARS-CoV-2 emergence, the multifaceted role of NETs on disease progression should be considered. As a detrimental factor during strong disease progression, NETs contribute negatively to severe pneumonia and acute respiratory distress syndrome (ARDS) after SARS-CoV-2 infection [39,40,41]. Patients suffer from silent hypoxia during the course of the disease due to impaired oxygen uptake. The fact that massive NET-formation is found in patients who suffer from severe hypoxia during viral infections also assumes that neutrophils are still fully functionally releasing NETs at those conditions. In addition, a direct role of HIF-1α on the pathogenesis due to upregulation and subsequent promoter activity was described [42]. The detrimental involvement of NETs in described diseases as ARDS, pneumonia, and COVID-19 highlights the urgent need to deepen the knowledge of the role of oxygen during NET formation. However, it remains to be determined what is the exact role and mode of action of NETs during SARS-CoV-2 infections. Our data show that NET formation can also occur under hypoxic conditions, which are a crucial factor during severe COVID-19. Taken together, this study highlights the importance of further basic research on the mechanisms behind the formation of neutrophil extracellular traps to gain a deeper understanding which finally could then help to develop improved treatment strategies which either make use of the mechanism of NET formation by boosting the host immune response or respectively to develop targets in NET formation pathways to keep an overshooting NET formation at bay, which would be of high relevance not only during the recent COVID-19 pandemic.

## 4. Materials and Methods

### 4.1. Transgenic Mice Breeding and Genotyping

In this study, we used a strain of mice that expressed a Cre-recombinase heterozygously, which cuts out the exon 2 at loxP sites in the *hif*-gene, thus leading to impaired protein function [24,25]. We confirmed the genotype of these mice via PCR. In short, tissue was incubated in lysis buffer at 60 °C until complete lysis. The lysate was centrifuged (17,000× *g*, 5 min), and the supernatant was transferred into a fresh tube with isopropanol, followed by further centrifugation. Isopropanol was decanted, and residues evaporated at 60 °C until no liquid was visible. The DNA was resuspended in ddH_2_O, incubated for 15 min at 60 °C, and frozen until usage for PCR. Genotypes were determined via PCR for the expression of loxP sites and Cre Recombinase (Primer, master mix, and thermal profiles: Supplementary Table S1).

### 4.2. Isolation of Murine Neutrophils from Bone Marrow

Bone marrow-derived neutrophils were isolated via negative selection using the EasySep™ Mouse Neutrophil Enrichment Kit from STEMCELL technologies (Vancouver, BC, Canada), according to manufacturer protocols. Beforehand, separated bones from hind legs, collected in buffer (RPMI with phenol red, 10% FCS, 1× Pen/Strep), were shortly washed in PBS and 3× in 70% EtOH before bone marrow from the tibia and femur was flushed, using a 26 G canula and a 10 mL syringe, with recommended medium (RPMI with phenol red, 10% FCS, 2 mM EDTA) through a 100 µm filter into a fresh falcon. After centrifugation (360× *g*, 4 °C, 7 min), erythrocytes were lysed by salt lysis with 10 mL of 0.2% NaCl for 20 s followed by the addition of 10 mL 1.6% NaCl. After the next centrifugation (360× *g*, 4 °C, 10 min), the supernatant was discarded, the pellet was resuspended in 1 mL STEMCELL media buffer (PBS, 2% FCS, 1 mM EDTA) and transferred to a fresh tube. Isolation of neutrophils was performed according to the kit’s protocol. Isolated cells were counted in a trypan blue solution with a hemocytometer. Cells from three individuals were pooled in 1 mL RPMI 1640 without phenol red (Gibco) after purification determination by flow cytometry, using Ly6G, Ly6C, and CD11b as a marker.

### 4.3. Purity Analysis via Flow Cytometry

Determination of purity was performed by analysis of cell-specific surface marker expression via flow cytometry. A combination of CD11b, Ly6G, Ly6GC, and respective isotypes, coupled with either FITC or PE was used for cell staining. 2 × 10^5^ cells were used per staining and incubated with either CD11b FITC and Ly6G PE, Ly6GC FITC, CD11b FITC diluted in STEMCELL media buffer for 45 min at 4 °C in the dark and subsequently filled up with 1 mL cold 1× PBS. Cells were then centrifuged at 360× *g* at 4 °C for 10 min. The supernatant was discarded, and the cells were resuspended in 250 µL RPMI for following flow cytometry at an Attune NxT Acoustic Focusing Cytometer (Life Technologies/Thermo Fischer Scientific, Waltham, MA, USA) at FSC 190, SSC 350, BL1 (FITC) 310 nm, BL2 (PE) 390 nm.

### 4.4. Neutrophil Isolation from Human Blood

Neutrophils were isolated from fresh blood, as previously described [43]. In short, fresh blood was drawn from healthy volunteers by a physician and directly used for isolation. Blood was layered 1:1 on Polymorphprep solution (Progen) in a 50 mL falcon tube and centrifuged at 472× *g* for 30 min at RT without brake. Afterward, the monocyte and plasma layer was removed, and with a fresh plastic Pasteur pipette, the neutrophil layer was transferred to a new falcon tube, directly filled up with 1× PBS at RT, and centrifuged at 472× *g* for 10 min with brake. The cell pellet was treated with 5 mL sterile H_2_O for erythrocyte lysis for 15 s and immediately filled up with 1× PBS, maximum 2 times. After another round of centrifugation at the same settings, the cell pellet was resuspended in 1 mL RPMI at RT. Cell count was determined as stated above.

### 4.5. Neutrophil Stimulation and Incubation under Normoxia and Hypoxia

Neutrophils were seeded in a density of 2 × 10^5^ cells per well in a 48-well suspension cell plate (Greiner bio-one) containing poly-L-lysin (0.01%, Sigma, St. Louis, MO, USA) coated glass coverslips. Cells were stimulated with either 25 nM Phorbol-12-myristat-13-acetate (PMA, Sigma), 10 mM Methyl-β-cyclodextrin (Sigma) for murine NET assays, 10 mM and 20 mM Methyl-β-cyclodextrin for human NET assays, Simvastatin 10 µM (Sigma), Mevastatin 50 µM (Sigma), or RPMI 1640 without phenol red as control, for 3 h under normoxia (18–21% O_2_) or hypoxia (1% O_2_) in a hypoxia glove box (COY Laboratories). After incubation, plates were shortly centrifuged for 5 min at 370× *g*, to bring down cells and NETs onto the coverslip. Finally, the cells were fixed with paraformaldehyde (PFA) at 4% final concentration for 15 min at RT and afterward stored at 4 °C until immune fluorescence staining.

### 4.6. Immune Fluorescence Staining and Confocal Laser Scanning Microscopy

Fixed coverslips were washed 3 times with 1× PBS to get rid of PFA and subsequently permeabilized with 0.5% TritonX100 for 5 min. Blocking was performed with 100 µL blocking buffer (PBS, 0.5% Tween20, 5% goat serum) for 20 min. Meanwhile, primary antibodies were prepared in blocking buffer. Incubation with primary antibodies, diluted in blocking buffer, against DNA/Histone complex (mouse monoclonal anti DNA/Histone1, Millipore, MAB 3864) and myeloperoxidase (rabbit anti-human myeloperoxidase, Dako, A0398) was done for 1 h at RT. After incubation, the samples were washed with 1× PBS and incubated with respective secondary antibodies (Alexa Fluor™ Plus 488, goat anti-mouse IgG, Invitrogen, A32723 and Alexa Fluor™ 633, goat anti-rabbit IgG, Thermo Scientific, Waltham, MA, USA, A21070), diluted in blocking buffer for 45 min at RT in the dark. Thereafter, the samples were washed with 1× PBS and mounted on ProlongGold antifade with DAPI (Thermo Fischer, Waltham, MA, USA) on a microscopy slide, which was sealed with nail polish the next day. Samples were analyzed via confocal microscopy with a Leica TCS SP5 confocal microscope with HCX PL APO 40× 0.75–1.25 oil immersion objective, using the 405 nm diode, 488 nm Argon, and 633 Helium lasers for excitation. Settings were adjusted in positive controls, and specificity was confirmed using isotype controls, whereafter settings were not changed anymore during the imaging session. Each sample was done in duplicate and imaged in total 6 times according to a predefined movement pattern across the sample slide.

### 4.7. Scanning Electron Microscopy

Neutrophils were harvested, seeded on glass cover slips, and stimulated with either RPMI, MβCD, or Simvastatin as stated above. After 3 h incubation under either hypoxia or normoxia, cells were fixed in 1.5% glutaraldehyde (Sigma-Aldrich Chemie GmbH, St. Louis, MO, USA) and 3% PFA, buffered with 0.1 M cacodylate buffer (Serva Electrophoresis, Heidelberg, Germany) for 24 h and subsequently washed with 0.1 M cacodylate buffer. For further processing, the samples were embedded with 1% osmium tetroxide (Science Services GmbH, Munich, Germany) and dehydrated in a series of graded ethanol, followed by critical-point-drying and coating with gold in a sputter-coater (SCD040, Oerlikon Balzers), as described previously [44,45] Afterward, the samples were mounted on 0.5” Aluminum Specimen Stubs (Agar Scientific, Stansted, Essex, UK) using 12 mm Leit-Tabs (Plano) and examined using a Zeiss EVO 15 scanning electron microscope (Carl Zeiss Microscopy, Oberkochen, Germany) operating with an acceleration voltage of 10 kV.

### 4.8. NET Analysis

NET images were counted manually by marking active and inactive cells on the image with ImageJ cell counter plugin (Version 1.51q). Cells were defined as active when they lost the lobular structure of the nucleus, were swollen, and showed positive staining for the respective NET markers mentioned in the method section. In addition, cells showing a distinct offshoot and cells touching these off shoots were considered active. The length, thickness, or structural appearance of extracellular fibers did not impact the quantification. Clearly, lobulated nuclei were marked as inactive. The rate of NET formation is given as a percentage in relation to the total amount of visible cells per image.

### 4.9. RNA Isolation and RT-qPCR

To analyse the expression of HIF-1α and its target gene *slc2a1*, RNA was isolated from bone marrow derived murine neutrophils of the aforementioned mice breed. Samples were centrifuged and solved in RLT buffer (Qiagen), including 1% of β-mercaptoethanol. For RNA extraction, the Qiagen RNease Mini Kit was used, according to the manufacturer’s instructions with the addition of a lysis step of 20× up and down pipetting of the sample through a 26 G canula at the beginning and a second elution step for a more efficient RNA yield at the end of the isolation.

The isolated RNA was tested for quality by 2100 Bioanalyzer (Agilent) with nano-chip according to manufacturer instructions. Subsequently, cDNA was transcribed via reverse transcription with High-Capacity cDNA Reverse Transcription Kit (Applied Biosystems, ThermoFischer) as stated in the protocol, except for a reaction size, increased to double amount. RT-qPCR was performed with 120 µL of cDNA for expression of HIF-1α exons2, as target of the aforementioned Cre-recombinase, exon 4, and exon 5 via exon spanning primers, as a control for genomic DNA contamination. Moreover, *slc2a1* expression was investigated as HIF-target gene. Expressions of said genes were normalized against expression of rps9 as HIF and hypoxia independent housekeeping gene (Primer, master mix and thermal profile stated in Supplementary Table S1). The RT-qPCR was performed in a AriaMx Real-time PCR System (Agilent), with automated threshold determination. ΔCt values were generated by subtraction of housekeeping gene Ct values from those of the target genes. Afterward, fold changes were determined by subtraction of ΔCt values of control conditions (HIF-1α exon4/5, normoxia) from ΔCt values from experimental condition (HIF-1α exon 2, hypoxia).

### 4.10. Oxygen Measurement during In Vitro Experiments

To ensure the application of hypoxic conditions during the experiment, extracellular oxygen levels were measured directly in the 48-well plates. Sensor spots (PreSens) were fixed with transparent silicon inside the wells and cells and media seeded as stated above. Oxygen levels were measured at 0, 60, and 180 min by positioning the sensor spots above a coaster connected to OXY1-ST (PreSens) measurement device, which was controlled via respective software from PreSens.

### 4.11. Neutrophil Stimulation under Hypoxia and Normoxia for Lipid Isolation and HPLC

Protocols were adapted and modified from Brogden et al., 2014, 2017 [46]. Freshly isolated neutrophils from murine bone marrow of WT and KO mice were seeded at a density of 1 × 10^6^ cells in 1.5 mL and incubated for 3 h at 37 °C, 5% CO_2_, similar to negative controls in NET stimulation assays. After incubation, samples were centrifuged at 400× *g* at 4 °C for 10 min. After washing with 1 × PBS, the cells were resuspended in 350 µL HPLC grade water and passaged through a 26 G canula 20 times for cell lysis. The lysate was filled with 1.6 mL HPLC grade water and transferred to glass tubes. 4 mL of methanol were given to the lysate, after 2 min 2 mL of chloroform was added and the tubes were rotated at RT for 30 min. Next, the tubes were centrifuged at 1147× *g* at 7 °C for 10 min. The supernatant was transferred to fresh glass tubes and filled up with 2 mL chloroform, shaken and further filled with 2 mL ddH_2_O. After phase formation, the tubes were shaken and again rotated for 10 min before centrifugation at same settings. The lipid containing methanol phase was taken to dry under nitrogen, to avoid oxidation of lipids, until no liquid was left and the precipitate was dissolved in 125 µL Acetonitril:Methanol for analysis via HPLC (VWR HITACHI Chromaster), against an external standard from 10 ng–500 mg/mL (Supplementary Figure S2). In detail, Hitachi High-Tech High Performance Liquid Chromatograph equipped with Chromaster UV 5410 detector was used. For cholesterol analysis, 10 μL of each sample were separated on a VDSpher PUR C18-H (3 μm, 150 × 2.0 mm) column (VDS Optilab, Berlin, Germany). The binary mobile phase consisted of 5 vol.% of 0.1% formic acid in H2O(A) and 95%ACN/MeOH (1:1, *v/v*) (B). Isocratic elution was performed at a flow rate of 0.2 mL/min and 12 °C column temperature. For measuring cholesterol, the area under the curve was determined and measured by comparing the samples peaks with the standard curve.

### 4.12. Statistical Analysis

For statistical analysis, GraphPad Prism 8 was used, as well as for graph and plot design. Significance was analyzed as stated in respective figure legends, after determination of Gaussian distribution. Data were given as mean with ±SD and differences are indicated via *p*-value (* *p* < 0.05, ** *p* < 0.01, *** *p* < 0.001, **** *p* < 0.0001).

### 4.13. Ethical Approval

Blood samples were drawn from healthy donors by a physician, in agreement with the local ethical board. The study was approved by ethical committee of the Hannover Medical School Nr. 3295-2016. Animal samples were acquired under approval by the ethical board of the University of Veterinary Medicine Hanover under TiHo-T-2020-13.

## Supplementary Materials

The following supporting information can be downloaded at: https://www.mdpi.com/article/10.3390/ijms23063195/s1.
