# Supplementary material for: Formation of Neutrophil Extracellular Traps by Reduction of Cellular Cholesterol Is Independent of Oxygen and HIF-1α
Source: Int J Mol Sci. 2022 Mar 16;23(6):3195. doi: 10.3390/ijms23063195 (PMC8954871; doi:10.3390/ijms23063195)
Supplement: Supplementary file 1 [file ijms-23-03195-s001.zip › ijms-1635023-supplementary.pdf]

Supplementary Material

Formation of Neutrophil Extracellular Traps by Cholesterol Depletion Is Independent of Oxygen and HIF1-α

Timo Henneck [1,2], AhmedElmontaser Mergani [1,2], Sabrina Clever [1,2], Anna E. Seidler [1,2], Graham Brogden [1,#], Sandra Runft [3], Wolfgang Baumgärtner [3], Katja Branitzki-Heinemann [1,2], Maren von Köckritz-Blickwede [1,2,*]

1 Department of Biochemistry, University of Veterinary Medicine Hannover
2 Research Center for Emerging Infections and Zoonoses (RIZ), University of Veterinary Medicine Hannover
3 Department of Pathology, University of Veterinary Medicine Hannover
* Correspondence: Maren.von.Koeckritz-Blickwede@tiho-hannover.de

Supplemental Material: Formation of neutrophil extracellular traps by cholesterol depletion is independent of oxygen and HIF1-α

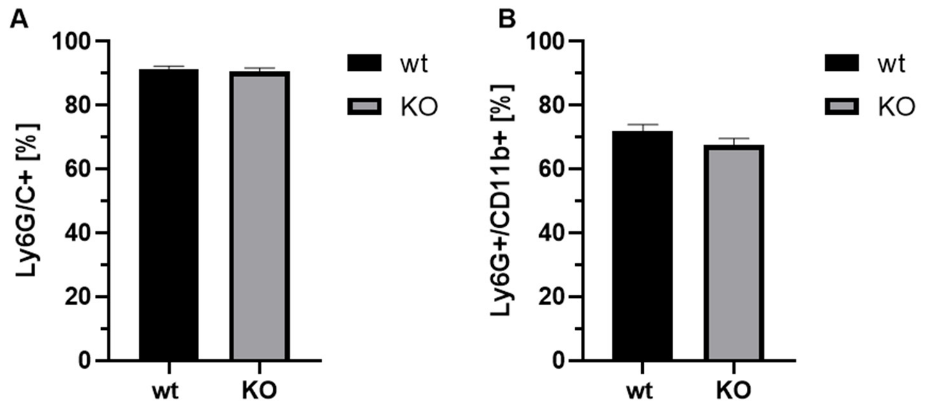

Figure S1. Identification of monocytic and granulocytic (A) and neutrophil (B) populations by Flow cytometry. The purity of the cell solution after the native selection was tested by separate incubation with FITC Rat anti mouse Ly6G/C (A) or PE Rat anti mouse Ly6G together with FITC Rat anti mouse CD11b (B) using FACS and after gating the singlets. (A) Both groups resulted in 90 % of monocytic and granulocytic singlet cells per animal. (B) Averaged 70 % of the cells were identified as matured neutrophils. Results represent mean with SEM

Table S1. Primer compositions and respective thermal profile of LyMCre PCR, Flox PCR and qPCR.

LysMCre PCR		
Agent	Volume (µl)	Final concentration
Primer LysMCre 1	1	0.3 µM
Primer LysMCre 2	1	0.3 µM
Primer LysMCre 3	0.5	0.15 µM
dNTPs	0.67	0.2 mM
10x Dream Taq buffer	3.33	0.2 U
dH_2O	24.67	
Dream Taq Polymerase	0.17	0.2 U/µl
Sample DNA	2	

Temperature (°C)	Length (s)	Cycles
95	120	1
95	30	35
60	30	
72	60	
72	600	1

4	∞	
FloxP PCR		
Agent	Volume (µL)	Final concentration
5' HIF-1α fl/fl	0.67	0.2 µM
3' HIF-1α fl/fl	0.67	0.2 µM
dNTPs	0.67	0.2 mM
10x Dream Taq buffer	3.33	
dH_2O	25.83	
Dream Taq Polymerase	0.17	0.2 U/µl
Sample DNA	2	

Temperature (°C)	Length (s)	Cycles
95	120	1
95	30	35
55	30	
72	60	
72	600	1
4	∞	

Real time qPCR	
Agent	Volume (µL)
SYBR green mix	10
Primer forward (10 µM)	1
Primer reverse (10 µM)	1
SIGMA-RNase free water	4

Temperature (°C)	Length (s)	Cycles
95	900	1
95	20	40
64	60	
72	20	
95	30	1
65	30	
95	30	

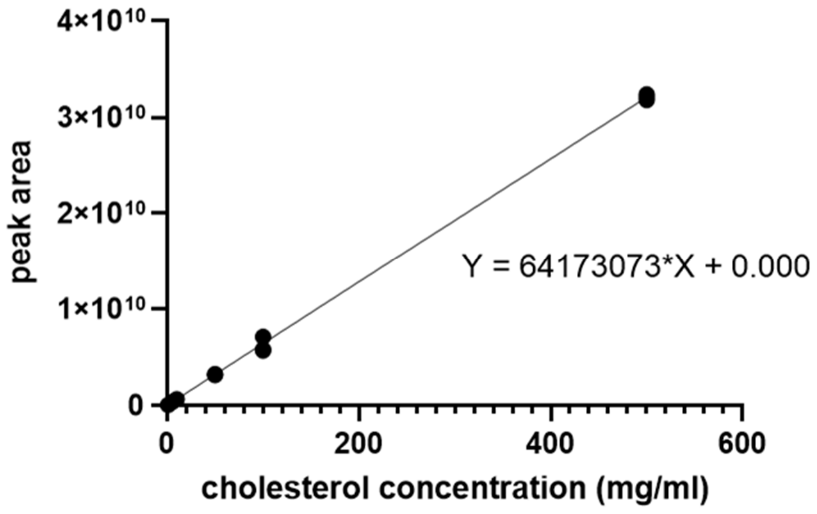

Figure S2. External cholesterol standard curve of 10 ng–500 mg/mL (n = 4).
